# Doxycycline-regulated gene expression in the opportunistic fungal pathogen *Aspergillus fumigatus*

**DOI:** 10.1186/1471-2180-5-1

**Published:** 2005-01-13

**Authors:** Keith Vogt, Ruchi Bhabhra, Judith C Rhodes, David S Askew

**Affiliations:** 1 Dept. of Pathology & Laboratory Medicine, University of Cincinnati College of Medicine, 231 Albert Sabin Way, Cincinnati, OH 45267-0529, USA

## Abstract

**Background:**

Although *Aspergillus fumigatus *is an important human fungal pathogen there are few expression systems available to study the contribution of specific genes to the growth and virulence of this opportunistic mould. Regulatable promoter systems based upon prokaryotic regulatory elements in the *E. coli *tetracycline-resistance operon have been successfully used to manipulate gene expression in several organisms, including mice, flies, plants, and yeast. However, the system has not yet been adapted for *Aspergillus spp*.

**Results:**

Here we describe the construction of plasmid vectors that can be used to regulate gene expression in *A. fumigatus *using a simple co-transfection approach. Vectors were generated in which the tetracycline transactivator (tTA) or the reverse tetracycline transactivator (rtTA2^s^-M2) are controlled by the *A. nidulans gpdA *promoter. Dominant selectable cassettes were introduced into each plasmid, allowing for selection following gene transfer into *A. fumigatus *by incorporating phleomycin or hygromycin into the medium. To model an essential gene under tetracycline regulation, the *E. coli *hygromycin resistance gene, *hph*, was placed under the control of seven copies of the TetR binding site (*tetO*_7_) in a plasmid vector and co-transfected into *A. fumigatus *protoplasts together with one of the two transactivator plasmids. Since the *hph *gene is essential to *A. fumigatus *in the presence of hygromycin, resistance to hygromycin was used as a marker of *hph *reporter gene expression. Transformants were identified in which the expression of tTA conferred hygromycin resistance by activating expression of the *tetO*_7_-*hph *reporter gene, and the addition of doxycycline to the medium suppressed hygromycin resistance in a dose-dependent manner. Similarly, transformants were identified in which expression of rtTA2^s^-M2 conferred hygromycin resistance only in the presence of doxycycline. The levels of doxycycline required to regulate expression of the *tetO*_7_-*hph *reporter gene were within non-toxic ranges for this organism, and low-iron medium was shown to reduce the amount of doxycycline required to accomplish regulation.

**Conclusions:**

The vectors described in this report provide a new set of options to experimentally manipulate the level of specific gene products in *A. fumigatus*

## Background

*Aspergillus fumigatus *is a saprophytic filamentous fungus that has become the leading mould pathogen in leukemia treatment centers and transplantation units in developed countries, second only to *Candida spp*. as a cause of systemic mycosis [1]. Despite some advances in therapy, currently available drugs for the treatment of aspergillosis continue to be hampered by problems with efficacy, toxicity, and the emergence of drug resistance. Moreover, a recent review of the *Aspergillus *case-fatality rate demonstrated that more than 50% of patients die with, or as a result of, aspergillosis, despite having received the reference standard of therapy [2]. The continued expansion of the immunosuppressed population emphasizes the need for increased understanding of both the basic biology and virulence of this mould so that more effective antifungal therapies can be developed.

The completion of the annotated sequence of the *A. fumigatus *genome is expected to greatly facilitate efforts to determine the contribution of specific gene products to the virulence of this opportunistic pathogen. Unfortunately, the genetic tractability of *A. fumigatus *has lagged behind some other fungal systems, particularly in the area of conditional expression systems. Inducible promoter systems have proven to be instrumental for the elucidation of gene function in a number of species, most notably with essential genes. Experimental manipulation of gene expression in *A. fumigatus *is presently accomplished through the use of DNA cassettes that are introduced into the organism as transgenes [3-5], inserted into specific chromosomal loci [3,6] or expressed from a multi-copy nonintegrating vector [7]. An inducible expression system based upon the ethanol-inducible *alcA *promoter from *A. nidulans *has been successfully used in A. *fumigatus *[8]. However, the conditions required to regulate the *alcA *promoter can have significant effects on the metabolism of the organism and thus remain a concern for many applications, particularly for *in vivo *studies.

**Figure 1:**
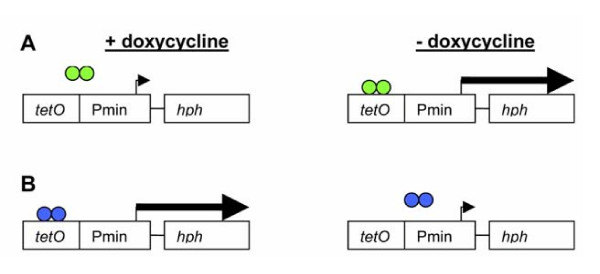
Schematic overview of the tetracycline-regulated gene expression system used in this study. The hygromycin resistance gene, *hph*, is under the control of seven copies of the TetR binding sequence, *tetO*, linked to a minimal promoter (Pmin). In the tTA-dependent expression system (A), the tTA protein (green circles) promotes hygromycin resistance of *A. fumigatus *by binding to the *tetO *promoter and activating transcription of the *hph *gene. Incorporation of doxycycline into the medium prevents tTA from binding to *tetO*, resulting in hygromycin sensitivity due to the absence of *hph *expression. The reverse tTA system (B) takes advantage of a reverse tetracycline transactivator, rtTA (blue circles), that binds to the *tetO *promoter only in the presence of doxycycline. In this case, rtTA promotes hygromycin resistance of *A. fumigatus *only when grown in the presence of doxycycline.

The tetracycline operator system has been used to regulate gene expression in a number of species. The system is based upon the *E. coli *tetracycline-resistance operon, a regulatory unit that detects minute concentrations of tetracycline and mounts an appropriate resistance response. Expression of the operon is controlled by a repressor protein, TetR that binds to operator sequences (*tetO*) in the promoter/enhancer region of the operon and prevents transcription. In the presence of tetracycline TetR is unable to bind *tetO*, which releases the repression and allows the operon to be expressed. This system has been adapted for experimental gene regulation in eukaryotes by fusing TetR to the VP16 transcriptional activating domain of herpes simplex virus VP16, thereby creating a synthetic tetracycline-regulatable transcriptional activator protein (tTA) that can be used to regulate a gene that is under the control of a tetracycline-responsive promoter (reviewed in [9] and shown schematically in Fig. 1). A tetracycline-regulated promoter is constructed by introducing one or more copies of the *tetO *sequence upstream of a minimal promoter region and the gene of interest (Fig. 1). In the absence of tetracycline, tTA is free to bind to the *tetO*-promoter and drive the expression of the downstream gene. The addition of tetracycline to the medium prevents tTA from binding the *tetO *sequences and the promoter is inactive. A variation of this system uses a 'reverse' tetracycline transactivator, rtTA that only binds *tetO *in the presence of tetracycline. In this case, a gene under *tetO *control is expressed in the presence of tetracycline, but not in its absence [10].

The TetR/*tetO *system is biologically active in a number of eukaryotes [11-13], including yeasts [14-17], but has not yet been adapted to the filamentous fungi. In this report we demonstrate that the tetracycline-regulated promoter system can be used to manipulate gene expression in *Aspergillus fumigatus *using a simple co-transfection procedure.

## Results

### Vector construction

**Figure 2:**
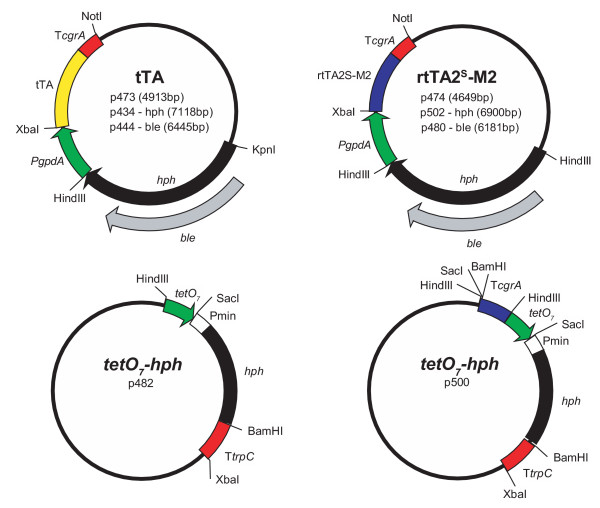
Schematic representation of plasmid constructions. The tTA gene is expressed from the *A. nidulans *promoter in three plasmids: p473 (no selection), p434 (hygromycin resistance encoded by *hph*) and p444 (phleomycin resistance encoded by *ble*). The rtTA2^S^-M2 gene is expressed from the *A. nidulans gpdA *promoter in p474 (no selection), p502 (hygromycin selection encoded by *hph*) and p480 (phleomycin resistance encoded by *ble*). Two versions of the *tetO*_7_-*hph *reporter construct are shown below. Plasmid p482 contains seven copies of *tetO*, a 175 bp minimal *gpdA *promoter (Pmin), and the *hph *gene encoding resistance to hygromycin. Plasmid p500 contains the same components, with the addition of a 280 bp fragment of the *A. fumigatus cgrA *terminator region upstream of the *tetO*_7 _promoter to reduce read-through from flanking sequences. All plasmids have been deposited in the Fungal Genetics Stock Center for distribution.

Details on plasmid construction are provided in Methods. The plasmids are shown schematically in Fig. 2 and the individual components are summarized in Table-1.

### Effects of doxycycline on the growth of A. fumigatus

**Figure 3:**
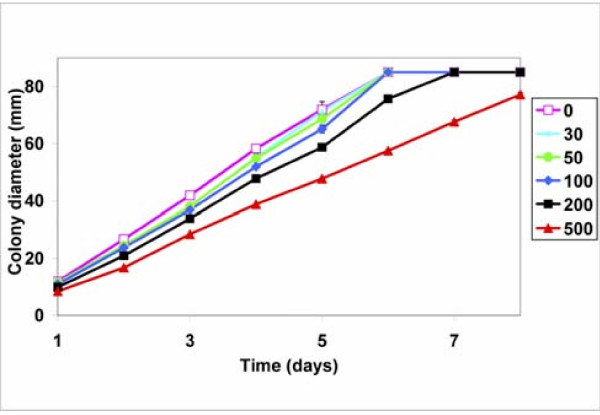
Sensitivity of *A. fumigatus *to doxycycline. Ten thousand conidia were spotted onto plates containing *Aspergillus minimal *medium and the indicated concentrations of doxycycline (μg/ml) and colony diameter was measured for 8 days at 37°C. This experiment used standard *Aspergillus *minimal medium adjusted to pH 6.5, but similar results were obtained when the pH was adjusted to 8 (data not shown).

Tetracyclines are small lipophilic antibiotics that readily diffuse into eukaryotic cells by passive diffusion. Doxycycline was selected for this study since it has the highest association equilibrium constant to TetR among the common tetracycline derivatives [18], and has been reported to be most effective in the regulation of tetracycline-regulated promoters in *S. cerevisiae *[19]. For the doxycycline system to be effective, the levels of doxycycline required to regulate a *tetO *promoter must not be within a toxic range for the organism. To determine the range of doxycycline concentrations that are tolerated by *A. fumigatus*, conidia were spotted onto the center of plates of *Aspergillus *minimal medium containing 0 – 500 μg/ml of doxycycline and colony diameter was measured with time. Concentrations up to 100 μg/ml had little effect on radial growth rates, all of which were within 5% of each other (Fig. 3). However, growth rate was reduced by 16% at 200 μg/ml and by 34% at 500 μg/ml of doxycycline. These results indicate that doxycycline can be used up to 100 μg/ml in minimal medium with no detectable effects on growth rate.

### Regulated expression of an essential gene by the 'tet-off' system

**Figure 4:**
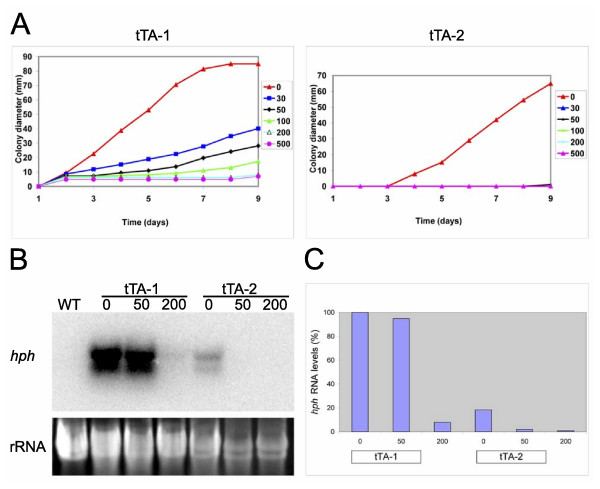
Effects of doxycycline on the hygromycin sensitivity of two *A. fumigatus *strains that express tTA (p444) in conjunction with the *tetO*_7_-*hph *reporter gene (p482). (A): Ten thousand conidia from the tTA-1 or tTA-2 transformants were spotted onto the center of a plate containing 1 mg/ml hygromycin and the indicated concentrations of doxycycline (μg/ml) and colony diameter was measured with time. (B): Doxycycline regulation of *hph *RNA levels in the tTA-1 and tTA-2 strains by Northern blot analysis. Total RNA isolated from overnight cultures grown in the presence of 0–200 μg/ml doxycycline was fractionated by agarose gel electrophoresis, transferred to nylon membranes, and probed with a ^32^P-labeled *hph *probe. (C): Hybridization intensity in each lane of the Northern blot in (B) was normalized to levels of the SYBR-green II-stained rRNA by phosphorimager analysis and is shown as a percentage of the levels seen in the tTA-1 transformant in the absence of doxycycline.

Inducible promoter systems are particularly useful for creating strains that can be inducibly depleted of an essential gene product [20]. To model an essential gene under *tetO *control we used heterologous expression of the *E. coli *hygromycin resistance gene, *hph*. The *hph *gene encodes a phosphotransferase that is essential to *A. fumigatus *in the presence of toxic concentrations of the aminoglycoside antibiotic hygromycin. The *hph *gene was cloned into a plasmid downstream of a hybrid promoter comprised of seven copies of the TetR binding sequence (*tetO*_7_) linked to a 175 bp minimal *gpdA *promoter from *A. nidulans *(p482, Fig. 2). The linearized *tetO*_7_-*hph *reporter plasmid was co-transfected into *A. fumigatus *protoplasts together with a linearized plasmid expressing the tetracycline transactivator, tTA (p444, Table-1) and transformants were selected on the basis of their resistance to hygromycin. Although p444 carries the *ble *gene, phleomycin selection was not included in this experiment. Thirteen hygromycin-resistant colonies were obtained from protoplasts transformed with the *tetO*_7_-*hph *reporter construct alone. Since these are integrative plasmids, the observed background colonies are presumed to be a consequence of positional effects at the site of integration, resulting in basal levels of expression of the *hph *reporter construct. By contrast, 192 colonies were obtained following co-transfection with p444 and the *tetO*_7_-*hph *reporter plasmid, suggesting that expression of tTA was driving expression of the *tetO*_7_-*hph *transgene and thus conferring hygromycin resistance. Fifty of these hygromycin-resistant colonies were randomly isolated and plated onto secondary hygromycin selection plates in the presence or absence of 100 μg/ml doxycycline. A total of five transformants showed increased hygromycin sensitivity in the presence of doxycycline, two of which were selected for further analysis: one showing marked hygromycin sensitivity in doxycycline (tTA-2) and one showing moderate hygromycin sensitivity (tTA-1). Conidia from each of these transformants were spotted into the center of a plate of minimal medium containing both doxycycline and hygromycin and the radial growth of the colony was monitored with time. The pH of the medium in this experiment was adjusted to 8 in order to maximize the hygromycin toxicity. As shown in Fig. 4A, the tTA-2 transformant showed tight regulation of the phenotype of hygromycin sensitivity. Doxycycline concentrations as low as 30 μg/ml completely arrested growth, indicating that a concentration of doxycycline that is inert to the growth of *A. fumigatus *(Fig. 3) can be used to modulate expression of an essential gene under *tetO *control in this fungus. Importantly, concentrations of doxycycline below 30 μg/ml could be used to manipulate the degree of hygromycin resistance; at 5 μg/ml and 2 μg/ml of doxycycline, the radial growth rate of the organism was reduced by 68% and 55%, respectively (data not shown).

Higher levels of doxycycline were required to suppress the growth of the tTA-1 transformant on hygromycin medium (Fig. 4A). Northern blot analysis showed that the tTA-1 strain expressed about 5-fold more *hph *RNA than tTA-2 (Fig. 4B), which was consistent with the fact that tTA-1 grew faster than tTA-2 in the presence of the same concentration of hygromycin (Fig. 4A, compare tTA-1 and tTA-2, no doxycycline). The doublet shown in Fig. 4B was occasionally seen on Northern blots hybridized to the *hph *probe and is presumed to represent alternative splicing of the primary *hph *transcript. The higher levels of *hph *RNA in the tTA-1 strain could be due to a combination of increased tTA expression (which would be expected to be susceptible to doxycycline regulation) and/or basal expression from one or more integrated copies of the *tetO*_7_-*hph *reporter gene (which would not be affected by doxycycline). Since there was a clear dose-response effect of doxycycline on *hph *expression and hygromycin resistant growth in this strain (Fig. 4A and 4B), it is likely that the two strains differ primarily in the amount of tTA that they express. Although Northern blot analysis showed barely detectable levels of tTA in either strain (data not shown), undetectable levels of tTA have been reported in other applications of the tetracycline regulatory system and are thought to be due to the toxic effects of overexpression [21]. Since very low levels of tTA protein are actually required to regulate a *tetO *promoter [21], even a small difference in tTA expression level that is beyond the limit of detection of a Northern blot could influence the amount of doxycycline required to suppress tTA activity in this transformant.

### Regulated expression of an essential gene by the 'tet-on' system

A limitation of the tTA-regulated system is that it requires inhibition of transcription rather than activation. To address this, a 'reverse' tTA has been generated (rtTA) that requires interaction of the transactivator with tetracyclines before *tetO *binding can occur, a system that is referred to as 'tet-on' [10]. Unfortunately, the mutations that reverse the response to doxycycline also reduce binding affinity for doxycycline ten-fold, thus requiring higher levels of doxycycline for maximal induction. Since there may be adverse effects associated with high doxycycline concentration in *A. fumigatus *under some conditions [22], we chose a derivative of rtTA that contains additional mutations that restore binding affinity for doxycycline [23]. One particular variant, rtTA2^S^-M2, also contains a multimerized minimal VP16 activation domain to enhance transcriptional activity, and its sequence has been manipulated to optimize expression in eukaryotic cells [23].

**Figure 5:**
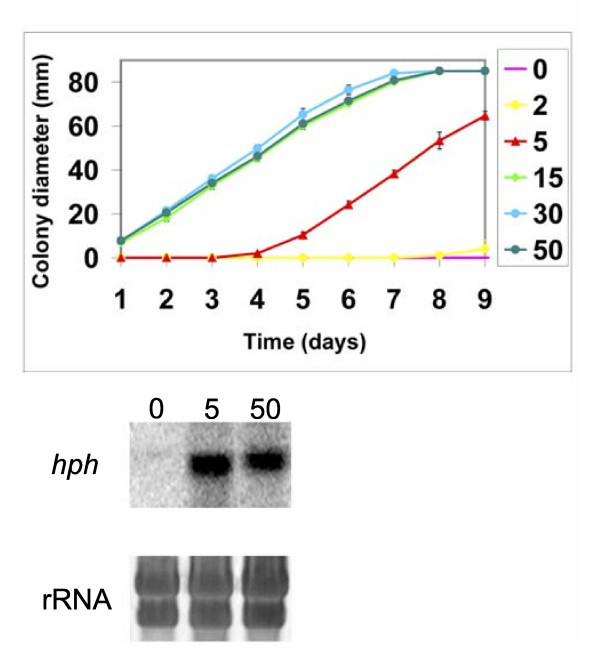
Effects of doxycycline on hygromycin sensitivity of a strain expressing rtTA in conjunction with the *tetO*_7_-*hph *reporter gene. Ten thousand conidia were spotted onto the center of a plate containing 750 μg/ml hygromycin and the indicated concentrations of doxycycline (μg/ml) and colony diameter was measured with time. Below: levels of *hph *RNA levels in the rtTA transformant by Northern blot analysis. Total RNA isolated from overnight cultures grown in the presence of 0, 5 or 50 μg/ml of doxycycline was fractionated by agarose gel electrophoresis, transferred to nylon membranes, and probed with a ^32^P-labeled *hph *probe.

Using the same co-transfection approach used for the tTA system, the *tetO*_7_-*hph *reporter (p500, Fig. 2) was co-transfected into *A. fumigatus *protoplasts together with a linearized plasmid that expresses rtTA2^S^-M2 (p474, Fig. 2) and the transformants were plated onto medium containing both hygromycin and doxycycline. In this experiment, a modified *tetO*_7_-*hph *reporter was used in which a 280 bp terminator sequence from the *A. fumigatus cgrA *gene [29] was inserted upstream of the *tetO*_7 _repeats to minimize read-through from flanking sequences (p500). Doxycycline was incorporated into the medium at 100 μg/ml to ensure that the *tetO*_7_-*hph *transgene would be expressed at sufficient levels to protect against hygromycin toxicity. Approximately 15% of 27 hygromycin resistant colonies showed reduced growth when shifted to hygromycin medium without doxycycline, one of which was selected for further analysis. As shown in Fig. 5, the inability of this transformant to grow in the presence of hygromycin was restored by the incorporation of as little as 5 μg/ml of doxycycline into the medium, indicating that low levels of doxycycline are biologically active as regulators of the *tetO *promoter in *A. fumigatus*. A further increase in hygromycin resistance was achieved at 15 μg/ml of doxycycline, but concentrations above 15 μg/ml had no additional effect. Northern blots analysis confirmed that the levels of *hph *RNA in the rtTA transformant were increased by the addition of doxycycline to the medium (Fig. 5). When hybridization intensity was normalized to SYBR-green II-stained rRNA bands by phosphorimager analysis, the levels of *hph *expression in the presence of both concentrations of doxycycline (Fig. 5) were thirty-fold greater than in the absence of added doxycycline.

### Doxycycline-regulation is enhanced by low- iron medium

**Figure 6:**
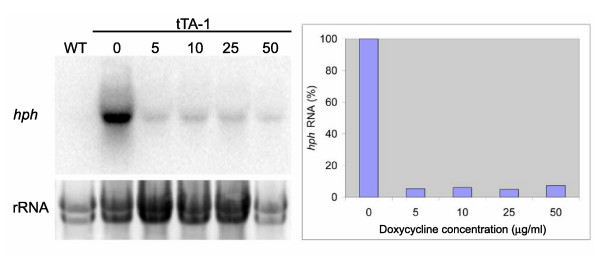
Doxycycline-regulation is enhanced in low-iron medium. Total RNA was isolated from an overnight culture of the tTA-1 transformant (Fig. 4) growing in *Aspergillus *minimal medium supplemented with one tenth the normal concentration of iron and the indicated concentrations of doxycycline (0–50 μg/ml). RNA was fractionated by agarose gel electrophoresis, transferred to nylon membranes, and probed with a ^32^P-labeled *hph *probe. Wild type *A. fumigatus *is included in the first lane as a negative hybridization control. Hybridization intensity was normalized to levels of SYBR-green II-stained rRNA by phosphorimager analysis and is presented in the graph as a percentage of the signal obtained in the tTA-1 strain in the absence of doxycycline (0).

A recent report has shown that iron blocks the accumulation and activity of tetracyclines in bacteria [24]. Since iron is a standard component of *Aspergillus *minimal medium, its presence may limit the efficiency of doxycycline-mediated gene regulation, particularly if transcriptional modulators with lower affinity for doxycycline are used. Fig. 6 shows the effects of lowering the iron concentration on doxycycline-mediated suppression of the *tetO*_7_-*hph *transgene in the tTA-1 clone showed in Fig. 4. In comparison to standard minimal medium, where 200 μg/ml of doxycycline was required to reduce expression in this strain (Fig. 4A and 4B), only 5 μg/ml was required in medium containing one tenth the normal concentration of FePO_4_·4H_2_0 (Fig. 6). This indicates that iron may also impair the accumulation of doxycycline in *A. fumigatus *and that the choice of medium could have significant effects on doxycycline-mediated gene regulation. Wild type *A. fumigatus *showed no reduction in radial growth rate on this low-iron minimal medium (data not shown).

## Discussion

The tetracycline-inducible method of gene regulation has become one of the most popular tools to manipulate gene expression in eukaryotes [25]. The efficacy of the system is attributed to the use of prokaryotic regulatory elements that respond to low concentrations of tetracyclines without affecting eukaryotic physiology, allowing control of gene expression without the concern for pleiotropic effects mediated by the effector. Although widely used in higher eukaryotes, including the model yeast *S. cerevisiae *[19], the system has not yet been reported in filamentous fungi. *Candida albicans *and *C. glabrata *are the only pathogenic fungi in which the system has been successfully applied thus far, however neither of these studies used the *tetR*-VP16 fusions upon which the tTA and rtTA systems are based [15-17].

In this study we show that both the tet-off (tTA) and tet-on (rtTA) systems can be used to regulate the expression of a hygromycin resistance reporter gene in *A. fumigatus*. Since the *hph *gene is essential in the presence of toxic levels of hygromycin, the ability to control hygromycin resistance by modulating the levels of *hph *transcription validates the system as a tool for analysis of essential genes in *A. fumigatus*. In the tTA system we found that individual transformants varied in the amount of doxycycline that was necessary to regulate expression of the *tetO*_7_-*hph *reporter gene. Since doxycycline prevents the tTA protein from binding to the *tetO *sequence, this is most likely due to variability in the amount of tTA protein that is expressed in each transformant. A limitation of the tTA approach described here is that the majority of the hygromycin-resistant transformants from the tTA/*tetO*_7_-*hph *co-transfection were not susceptible to regulation by doxycycline. This may be due in part to leaky expression of the *tetO*_7_-*hph *reporter, caused by enhancers in the proximity of the integration site [21,25]. A second possibility is that the levels of tTA coming from the *gpdA *promoter used in this study were too high to be removed by non toxic concentrations of doxycycline. Since lower levels of tTA expression are more readily suppressed by doxycycline, it is conceivable that a weaker promoter used to drive tTA would increase the frequency with which doxycycline-regulatable transformants can be isolated. Lower levels of tTA expression could also be accomplished by using a shorter segment of the *gpdA *promoter used in this study.

The ability to quantitatively control expression from the *tetO*_7_-*hph *reporter gene was also observed in a strain expressing the reverse transactivator, rtTA. Concentrations of doxycycline from 2 μg/ml to 15 μg/ml gave a graded response of hygromycin resistance, indicating that *A. fumigatus *is responsive to concentrations of doxycycline that are similarly effective in *S. cerevisiae *[19] and *C. albicans *[15]. Moreover, this level of sensitivity falls within the range of doxycycline concentrations that can be achieved in mouse tissues [15,16], raising the possibility of using this system to modulate the expression of virulence-related genes in pathogenesis studies on *A. fumigatus*. Only 15% of the hygromycin-resistant colonies from an rtTA/*tetO*_7_-*hph *co-transfection showed doxycycline-dependent hygromycin resistance however, suggesting that some of the hygromycin resistance was due to leaky expression of the *tetO*_7_-*hph *gene. Leakage of *tetO*_7_-regulated genes has been described in other systems, and is attributed to enhancers located in the proximity of the integration site that increase expression of the *tetO*-linked gene [21,25]. This type of problem will affect *tetO*_7_-controlled genes regardless of whether they are integrated randomly in the genome or targeted to specific loci.

## Conclusions

This report establishes the utility of the tetracycline-regulated system as an approach to regulate gene expression in *A. fumigatus*. A limitation of the system was that only 10–15% of the transformants could be regulated by doxycycline, either when tTA or rtTA were used, emphasizing the need to screen for regulatable transformants. A recent approach to limit the problem of leakiness of a *tetO*-driven gene is the use of trans-silencer proteins comprised of fusions between *tetR *and a transcriptional silencing domain [26,27]. It is conceivable that the incorporation of a synthetic *A. fumigatus*-derived trans-silencer protein into the co-transfection approach described in this study would improve the efficiency of the system.

## Methods

### Vector construction

All vectors are based on the pBluescript plasmid (Stratagene) and were linearized prior to transfection. PCR amplification of components were performed using standard amplification protocols using PfuTurbo DNA polymerase (Stratagene).

#### Hph Reporter Constructs *(p482 and p500)*

A segment containing seven copies of the *tet *operator sequence (*tetO*_7_) was PCR amplified from pUHD10-3 [12] with the forward primer 5'-aagcttgcgtatcacgaggccctttc and the reverse primer 5'-aagcttctcgacccgggtaccgag (added *Hin*dIII cloning sites are underlined) and cloned into the *Hin*dIII site of pBluescript. A 1.6 kb fragment containing a minimal *gpdA *promoter from *A. nidulans *(-175 relative to the ATG of the *hph *open reading frame), the *hph *gene encoding resistance to hygromycin, and the *trpC *terminator from *A. nidulans*, was then PCR amplified from pAN7-1 [28] with forward primer 5'-gagctccccatcttcagtatattcatc (added *Sst*I cloning site underlined) and reverse primer 5'-tctagatcgcgtggagccaagagcgg (added *Xba*I cloning site underlined) and cloned downstream of *tet0*_7 _into the *Sst*I and *Xba*I sites of the plasmid, creating p482. To minimize read-through from flanking sequences into *tet0*_7_, a 280 bp segment of the terminator region of *A. fumigatus cgrA *[29] was inserted upstream of *tet0*_7 _PCR to create p500. The *cgrA *terminator was PCR amplified from genomic DNA of *A. fumigatus *isolate H237 using the forward primer 5'aagcttacagcagaagaatctctc (added *Hin*dIII cloning site underlined) and reverse primer 5'ctcgagatgattcatgacgtatattc (added *Xho*I cloning site underlined), cloned into pCR2.1-Topo (Invitrogen), excised with *Hin*dIII, and inserted upstream of *tetO*_7 _in p482 to create p500.

#### tTA expression vectors (p473, p434, and p444)

A segment of the *A. nidulans gpdA *promoter was amplified from pAN7-1 [28] (position -679 to -1, with +1 being the start of the *hph *open reading frame) using the forward primer 5'-aagcttcggagaatatggagctt (added *Hin*dIII cloning site underlined) and the reverse primer 5'-gaattcggtgatgtctgctcaag (added *Eco*RI cloning site underlined) and cloned into pBluescript at the same sites. The tTA gene was then PCR amplified from pUHD15-1 [12] with the forward primer 5'-gaattctggcaatgtctagattagataaaag (added *Eco*RI cloning site underlined) and reverse primer 5'-atcatgtctggatcctcgcg (internal *Bam*HI site underlined) and cloned into the *Eco*RI and *Bam*HI sites downstream of the *gpdA *(-679) promoter. A 280 bp segment of the terminator region of *A. fumigatus cgrA *[29] was then amplified from H237 genomic DNA using the forward primer 5'-actagtacagcagaagaatctctc (added *Spe*I site underlined) and reverse primer 5'-gcggccgcatgattcatgacgtatattc (added *Not*I site underlined) and inserted into the *Spe*I and *Not*I sites downstream of tTA. To introduce phleomycin selection into this construct, a phleomycin resistance cassette containing the *A. nidulans gpdA *promoter, the *Streptoalloteichus hindustanus ble *gene encoding resistance to phleomycin, and the *S. cerevisiae CYC1 *terminator was amplified from pBCphleo (Fungal Genetics Stock Center) using the forward primer 5'-cctcaggcggagaatatggagcttcatcg and the reverse primer 5'-cctcaggaattaaagccttcgagcgtccc. The PCR product was cloned into pCR-Blunt II-TOPO (Invitrogen), excised with *Kpn*I and *Xho*I and inserted into the P*gpdA*-tTA construct to create p444. The phleomycin cassette was excised from p444 with *Hin*dIII and re-ligated to create p473. To introduce hygromycin selection into p444, the phleomycin cassette was excised with *Kpn*I and *Hin*dIII and replaced with a hygromycin resistance cassette (containing the *A. nidulans gpdA *promoter, the *hph *gene encoding resistance to hygromycin, and the *trpC *terminator from *A. nidulans*) that was amplified from pAN7-1 [28] with forward primer 5'-ggtacccggagaatatggagcttc (added *Kpn*I cloning site underlined) and reverse primer 5'-aagcttgcttgagagttcaaggaag (added *Hind*III cloning site underlined) to make p434.

#### rtTA expression vectors

The tTA gene was excised from p473 with *Eco*RI and *Bam*HI and replaced with an *Eco*RI-*Bam*HI fragment containing the rtTA2^s^-M2 variant of rTA from pUHrT62-1 (generous gift from C. Berens, Erlangen, FRG) to create p474. To introduce phleomycin resistance into p474, the phleomycin resistance cassette was excised from p444 with *Kpn*I and *Hind*III and cloned into the same sites in p474 to create p480. To introduce hygromycin resistance into p474, the hygromycin resistance cassette described in p434 was excised from an unrelated plasmid as a *Hin*dIII fragment and cloned into the *Hin*dIII site of p474 to make p502.

### Strains and culture conditions

The *A. fumigatus *strains used in this study are listed in Table-1. The wild-type strain, H237, is a clinical isolate. Conidia were harvested from strains grown on *Aspergillus *minimal medium plates [30]. This minimal medium contains 4.5 μM FePO_4_·4H_2_0. For low-iron minimal medium, the FePO_4_·4H_2_0 concentration was reduced to 0.45 μM.

Plasmids were introduced into *A. fumigatus *protoplasts as previously described [3]. Following transformation, protoplasts were plated onto 20 ml of osmotically stabilized minimal medium containing 100 μg/ml doxycycline (for transformations involving rtTA-expressing plasmids) or no added doxycycline (for transformations involving tTA-expressing plasmids). After incubating at room temperature overnight, each plate was overlaid with 10 ml of minimal medium top agar containing 0.5% agar, 1M sorbitol, and 8 mg hygromycin B (Invivogen, San Diego, CA). Doxycycline was also incorporated into the top agar overlay (100 μg/ml) for experiments involving rtTA-expressing plasmids. Colonies arising on these primary plates were transferred onto secondary selection plates containing the same selective agents, and conidia from the secondary plates were replated onto selective medium at low density to isolate colonies derived from single conidia. All subsequent experiments were performed on monoconidial isolates. For co-transfection experiments, 5 μg of the linearized *tetO*_7_-*hph *reporter construct was co-transfected with 5 μg of the linearized tTA plasmid (p444), or 50 μg of the linearized rtTA plasmid (p474).

For experiments addressing the effects of doxycycline on hygromycin sensitivity, ten thousand conidia were spotted onto the surface of *Aspergillus *minimal medium agar containing hygromycin and doxycycline at the concentrations specified in the Figure legends. The plates were then incubated at 37°C, and colony diameter was measured with time. Radial growth rates were calculated from the exponential part of the resulting growth curves.

### Northern blot analysis

For analysis of *hph *gene expression, RNA was isolated from overnight cultures in minimal medium supplemented with the indicated concentrations of doxycycline by crushing in liquid nitrogen and extracting RNA from the crushed mycelium with phenol/chloroform. Twenty micrograms of total RNA were fractionated by formaldehyde gel electrophoresis as previously described [20], transferred to positively charged nylon membranes (MSI, Inc., Westborough, MA, USA) and hybridized to a ^32^P-labeled *hph *DNA probe under stringent conditions in 50% (v/v) formamide/5XSSC (1X SSC is 0.15 M NaCl/0.015 M Na_3_·citrate, pH 7.6)/2X Denhardt's solution/10% (w/v) dextran sulfate/1% (w/v) sodium dodecyl sulfate (SDS). The *hph *probe was an 800 bp *Eco*RI-*Bam*HI fragment from pAN7-1 [28] containing a segment of the *hph *open reading frame. Hybridization intensity was quantified with a Phosphorimager (Molecular Dynamics) and normalized for differences in gel loading by quantitating the relative levels of SYBR-green II-stained rRNA (Molecular Probes, Inc., Eugene, OR, USA).

## List of abbreviations

tTA tetracycline transactivator

rtTA reverse tetracycline transactivator

TetR tetracycline repressor

*tetO *TetR binding sequence

*hph *hygromycin resistance gene

*ble *phleomycin resistance gene

## Authors' contributions

KV participated in vector construction, gene transfer into *A. fumigatus*, screening of transformants and drafting the manuscript. RB participated in plasmid construction. JCR contributed to the planning of the study. DSA conceived of the project and directed its design and execution. All authors have read and approved the final manuscript.

## Figures and Tables

**Table 1:** Plasmids and strains

**Plasmids**	**Selectable marker**			**Transactivator**		
	**Promoter**	**Gene**	**Terminator**	**Promoter**	**Gene**	**Terminator**

p473	-	-	-	*gpdA*(-679)	tTA	*cgrA*
p434	*gpdA*(-679)	*hph*	*trpC*	*gpdA*(-679)	tTA	*cgrA*
p444	*gpdA*(-679)	*ble*	*CYC1*	*gpdA*(-679)	tTA	*cgrA*
p474	-	-	-	*gpdA*(-679)	rtTA	*cgrA*
p502	*gpdA*(-679)	*hph*	*trpC*	*gpdA*(-679)	rtTA	*cgrA*
p480	*gpdA*(-679)	*ble*	*CYC1*	*gpdA*(-679)	rtTA	*cgrA*
p482	*tetO*_7_-*gpdA*(-175)	*hph*	*trpC*	-	-	-
p500	T*cgrA*-*tetO*_7_-P*gpdA*(-175)	*hph*	*trpC*	-	-	-

**Strains**	**Genotype/construction**			**Source**		

wt	H237			David Holden		
Af-tTA-1	H237 (p444, p482) – isolate 1			This study		
Af-tTA-2	H237 (p444, p482) – isolate 2			This study		
Af-rtTA	H237 (p480, p500)			This study		
